# Recent Advances in Metal-Free Quinoline Synthesis

**DOI:** 10.3390/molecules21080986

**Published:** 2016-07-29

**Authors:** Ginelle A. Ramann, Bryan J. Cowen

**Affiliations:** Department of Chemistry and Biochemistry, University of Denver, Denver, CO 80208, USA; ginelleramann@gmail.com

**Keywords:** quinoline, heterocycle, metal-free

## Abstract

The quinoline ring system is one of the most ubiquitous heterocycles in the fields of medicinal and industrial chemistry, forming the scaffold for compounds of great significance. These include anti-inflammatory and antitumor agents, the antimalarial drugs quinine and chloroquine, and organic light-emitting diodes. Quinolines were first synthesized in 1879, and since then a multitude of synthetic routes have been developed. Many of these methods, such as the Skraup, Doebner–Von Miller, and Friedlander quinoline syntheses, are well-known but suffer from inefficiency, harsh reaction conditions, and toxic reagents. This review focuses on recent transition metal-free processes toward these important heterocycles, including both novel routes and modifications to established methods. For example, variations on the Skraup method include microwave irradiation, ionic liquid media, and novel annulation partners, all of which have shown increased reaction efficiency and improved yield of the heteroring-unsubstituted quinoline products. Similarly, modifications to other synthetic routes have been implemented, with the quinoline products displaying a wide variety of substitution patterns.

## 1. Introduction

**Figure 1:**
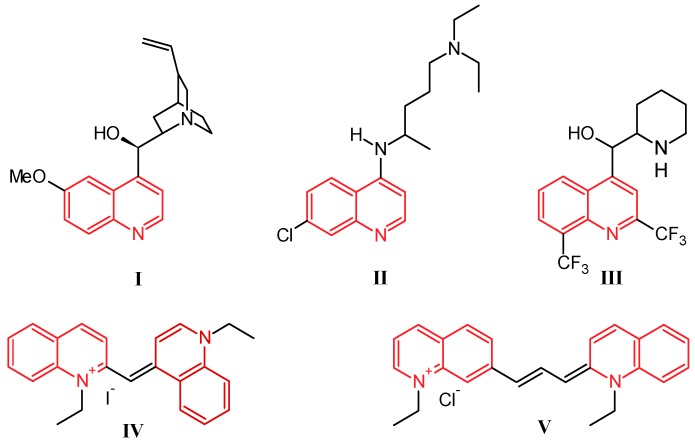
Structures of quinine (**I**), chloroquine (**II**), mefloquine (**III**), ethyl red iodide (**IV**), and pinacyanol (**V**).

Quinoline was discovered by Runge in 1834 as one of the many components extracted from coal tar [1]. While this nitrogen-based heterocycle is not overly useful in and of itself, it is easily modified with simple to complex functionalities, giving a multitude of compounds that are ubiquitous in the fields of medicinal and industrial chemistry. Prominently, the first and most widely used antimalarial agent, quinine (**I**, Figure 1), contains the quinoline scaffold, as do the closely related derivatives chloroquine (**II**) and mefloquine (**III**) [2]. Several promising anti-inflammatory and antitumor therapeutics are also built on this structure. Alternatively, quinolines provide frameworks for industrial uses including organic light-emitting diodes (OLEDs) and photovoltaic cells, as well as solvents for terpenes and resins. In addition, quinoline-based dyes such as ethyl red iodide (**IV**) and pinacyanol (**V**) have been used since the beginning of the nineteenth century in photographic plates [3].

The prevalence of the quinoline ring system in a vast range of medical and industrial settings can be ascribed mainly to its versatility and broad potential for functionalization. In fact, it is this versatility which earned it the designation of “privileged scaffold” in medicinal chemistry, a term coined by Evans in 1988 which refers to simple structural subunits present in diverse therapeutic compounds with distinctive receptor affinities [4]. Consequently, the synthesis of variously substituted quinolines has been a recurring endeavor for nearly a century and a half [5]. A multitude of synthetic methods have been established over this timeframe, which construct the quinoline ring from diverse starting materials and result in products with nearly limitless combinations of functionality.

## 2. Established Methods of Quinoline Synthesis

**Figure 2:**
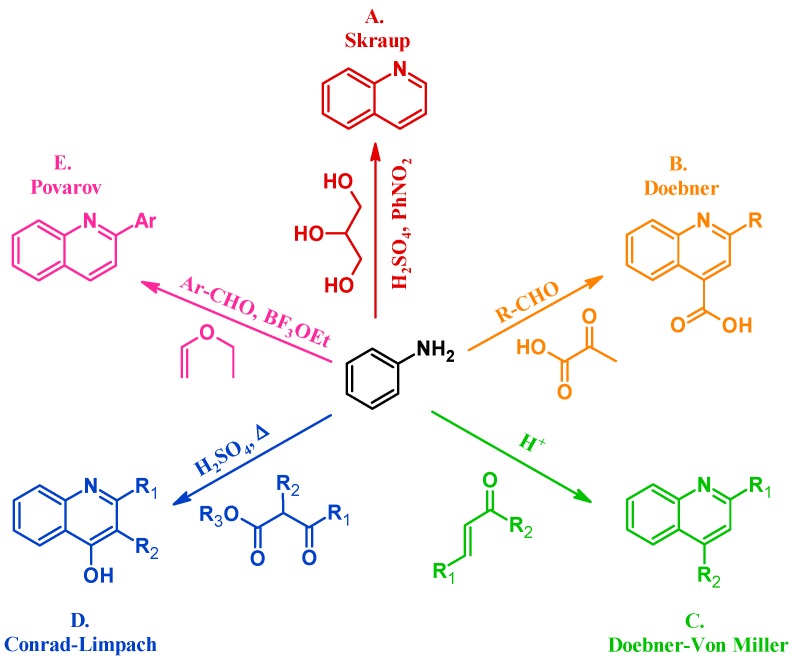
Quinoline synthesis methods: Skraup reaction (**A**); Doebner reaction (**B**); Doebner–Von Miller reaction (**C**); Conrad–Limpach reaction (**D**); and Povarov reaction (**E**).

Many methods have been instituted to react aniline with various annulation partners to obtain quinolines. One of the oldest and most enduring of such procedures is the Skraup reaction (A, Figure 2). In 1880, Czech chemist Zdenko Hans Skraup heated aniline and glycerol with an oxidant in concentrated sulfuric acid, a method that is still commonly used for the production of heteroring-unsubstituted quinolines [6]. The Doebner method (B), introduced by Oscar Doebner in 1887, combines aniline with an aldehyde and pyruvic acid to give 2-substituted quinoline-4-carboxylic acids. A variation of the Skraup procedure, the Doebner–Von Miller reaction (C) was introduced in 1881 and uses α,β-unsaturated aldehydes or ketones to obtain 2- and 4-substituted quinolines under acidic conditions. The Conrad–Limpach reaction (D), reported in 1887, is also conducted by refluxing in acid, but uses β-ketoesters as the annulation partner to give 2- and 3-substituted quinolin-4-ols [7]. As a contemporary example, in 1963 L.S. Povarov and coworkers reacted aryl aldehydes and activated alkenes to form 2-arylquinolines (E) [8].

**Figure 3:**
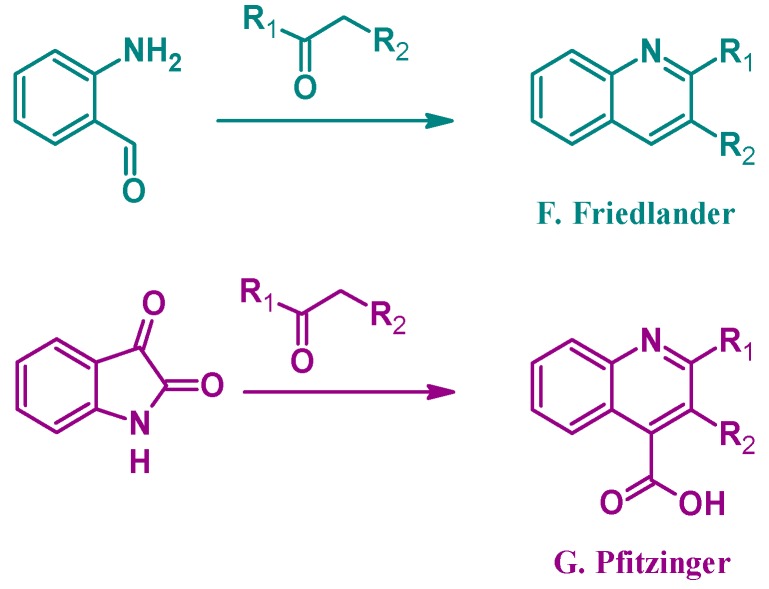
Quinoline synthesis methods: Friedländer reaction (**F**); and Pfitzinger reaction (**G**).

Alternatively, several other established methods of quinoline synthesis utilize substrates other than aniline in the cyclization process. The Friedländer reaction (F, Figure 3), reported by Paul Friedländer in 1882, uses 2-aminobenzaldehyde with another carbonyl component to form 2- and 3-substituted quinolines. Similarly, the Pfitzinger (G) reaction combines isatin with a carbonyl compound under basic conditions to give 2- and 3-substituted quinoline-4-carboxylic acids [9]. While not an exhaustive list, these examples highlight the breadth of options in the formation of quinoline ring systems.

In general, these methods are powerful tools in the synthesis of a broad range of quinolones-from unsubstituted to highly functionalized. However, none of the methods described thus far are without drawbacks. Many suffer from low efficiency, harsh reagents and reaction conditions, and functional group incompatibility. Therefore, the quest to modify these reactions to address their inherent issues has been a recurring endeavor of numerous research groups.

This review documents recent progress in quinoline synthesis, particularly in the area of transition metal-free methods. While transition metal catalysis has been a popular field of late, those reactions are outside the scope of the current paper and will not be covered.

The review is organized into two main sections. First, established procedures will be addressed, highlighting the problems unique to the individual procedures and detailing modifications in recent literature. Then novel reaction schemes will be explored and compared to previous examples.

## 3. Quinoline Synthesis via Modifications to Aniline-Based Established Methods

### 3.1. Skraup Reaction

As previously mentioned, the Skraup reaction is one of the oldest methods of quinoline synthesis and is still widely employed in the formation of heteroring-unsubstituted quinolines. However, the conventional reaction outlined by Skraup, in which aniline and glycerol are refluxed with an oxidant for an extended period of time in concentrated sulfuric acid, ubiquitously results in a thick tar from which the crude product is difficult to extract. The harsh reaction conditions, toxic reagents, and low yield of product do not recommend this procedure for practical application. Nonetheless, a dearth of procedures that are applicable to formation of heteroring-unsubstituted quinolines exist, compelling several groups to modify the reaction conditions to mitigate these inherent flaws.

**Figure 4:**
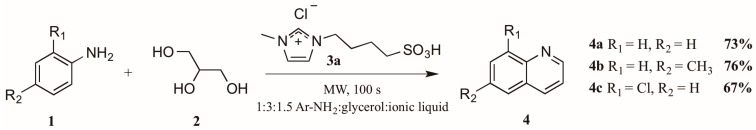
Skraup reaction in ionic liquid medium under microwave irradiation.

The use of microwave irradiation in place of conventional heat sources has become popular over the last decade. Cristea et al. [10] found microwave irradiation to be an efficient heat source for the synthesis of quinolines, reducing the required reaction time and increasing yield. However, their continued use of arsenic pentoxide as oxidizing agent did not ameliorate the harsh reaction conditions common to this method. Drawing on this work, Amarasekara and Hasan [11] also applied microwave heating to the Skraup synthesis, reacting anilines **1** with glycerol **2** in a 1:3 ratio (Figure 4). Divergently, replacement of the concentrated sulfuric acid with an imidazolium cation-based sulfonic acid ionic liquid **3a** significantly improved the reaction outcome; it was also noted that addition of an exogenous oxidant was unnecessary. Using these modifications, they obtained quinoline product **4** in very good yields.

**Figure 5:**
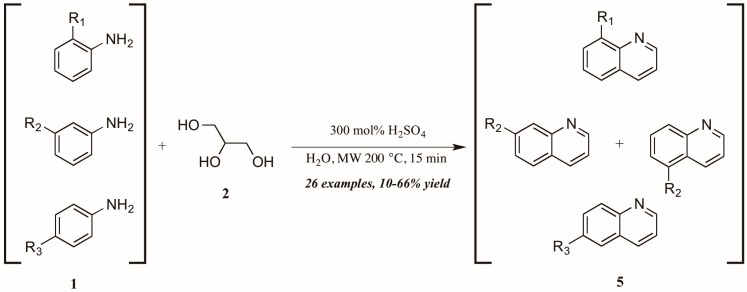
Skraup reaction under microwave irradiation.

Concurrently—and also citing Cristea and coworkers as foundational—Len et al. [12] applied microwave heating to quinoline synthesis within their laboratory′s theme of “green chemistry” techniques. Omitting the exogenous oxidant and heating monosubstituted anilines **1** with glycerol **2** in concentrated sulfuric acid under 200 °C microwave irradiation, they obtained a wide variety of mono-functionalized quinoline analogs **5** in fair to good yield (Figure 5). They also noted the essentiality of sulfuric acid as catalyst and the improved extraction efficacy with the addition of water to the reaction mixture as solvent.

**Figure 6:**
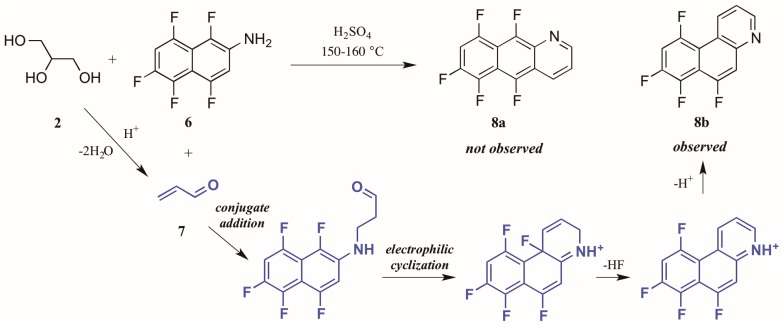
Electrophilic substitution of fluorine in the Skraup reaction.

Shteingarts and coworkers [13] found an interesting divergence of reactivity during their investigation of biologically-relevant polyfluorinated heterocycles. Refluxing 1,4,5,6,8-pentafluoro-2-naphthylamine **6** with glycerol **2** in H_2_SO_4_, this group expected to see cyclization at the unsubstituted *ortho* position. However, to their surprise they isolated only the polyfluorinated benzo[*f*]quinoline, a product of electrophilic substitution of fluorine in the 1-position (Figure 6). The proposed mechanism for this annulation begins with the conjugate addition of naphthylamine to acrolein **7**, the product of glycerol dehydration and generally presumed to be the active annulation partner in the Skraup reaction [6]. Cyclization then occurs through attack of the aromatic ring on the carbonyl group. Then after tautomerization and loss of HF, the ring is rearomatized, forming the observed product **8b**.

### 3.2. Doebner Reaction

The Doebner reaction, while not as well-known as the Skraup, is another method of forming quinoline rings from simple starting materials. The general procedure, combining aniline with benzaldehyde and pyruvic acid to form 2-substituted quinoline-4-carboxylic acids, typically suffers from low yields or long reaction times. Even previous examples of modified procedures require the use of harsh reaction conditions, large amounts of organic solvent, or special apparatus [14].

**Figure 7:**
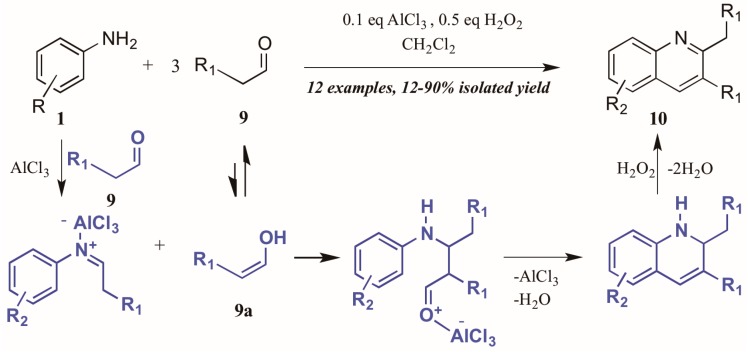
Doebner reaction using hydrogen peroxide as oxidant with Lewis acid catalyst.

Guo et al. [15] approached quinoline synthesis via a Doebner-like process, in which they first condensed aniline **1** with an aldehyde **9** (Figure 7). In contrast to the typical method, they replaced the pyruvic acid component with a second molecule of aldehyde, thus obtaining quinolines substituted at the 2- and 3-positions. Moreover, they found that addition of a substoichiometric amount of H_2_O_2_, acting as an environmentally-friendly oxidant alternative, increased the yield of quinoline product, as did addition of a catalytic amount of aluminum chloride. The proposed mechanism of this annulation begins with the AlCl_3_-mediated condensation of the aniline with one molecule of aldehyde to give the imine. The enol tautomer of a second molecule of aldehyde (**9a**) then adds to the imine, which cyclizes to give the tetrahydroquinoline. Loss of one equivalent of H_2_O gives the dihydroquinoline, which then aromatizes under hydrogen peroxide oxidation to give the final product.

**Figure 8:**
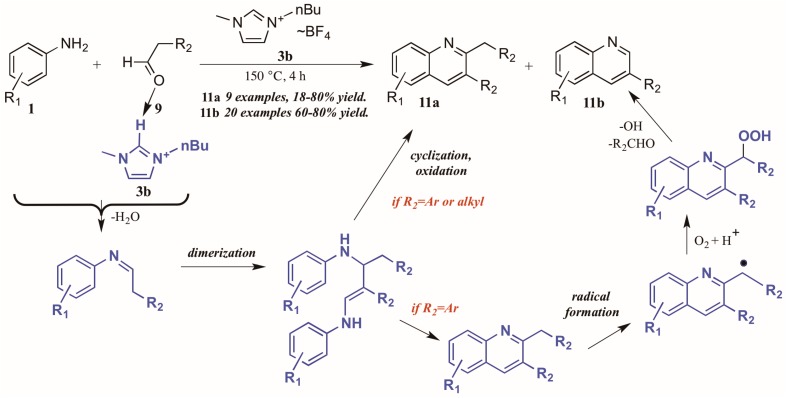
Divergent reactivity in the Doebner reaction in ionic liquid medium.

Bharate and Vishwakarma [16] also reported a method toward 2,3-disubstituted and 3-substituted quinolines using anilines and aldehydes (Figure 8). Drawing on previous work by Yan et al., which used copper(I) bromide and trifluoromethanesulfonic acid in DMSO in the formation of these heterocycles, they found that omission of the transition metal and replacement of the acid and solvent with an ionic liquid ([Bmim]BF_4_, **3b**) gave comparable yields of the two products in their model reaction.

Interestingly, investigations into the scope of the reaction indicated that the ratio of mono-to-disubstituted product was a direct result of both reaction time and the nature of the aldehyde substrate. Using phenylacetaldehyde substrates, nearly equivalent amounts of the two products could be achieved by a 30 min reaction time, while extending the time to 4 h produced monosubstituted quinoline as the sole product. However, aliphatic aldehydes gave only the disubstituted quinoline.

This phenomenon was rationalized through mechanistic inquiry. The ionic liquid reaction medium is presumed to play an integral part by hydrogen bonding with the aldehyde, thus increasing its electrophilicity. The aniline **1** attacks the aldehyde **9**, forming an imine through loss of H_2_O. This imine, acting through its enamine tautomer, self-condenses into a dimer. This dimer then cyclizes, extruding the starting aniline, then oxidizes to the disubstituted product **11a**. If the R_2_ group is aryl, a benzyl radical could then form, possibly with assistance from the ionic liquid medium. This radical then reacts with molecular oxygen to form the peroxy acid. This peroxy acid is extruded as benzaldehyde and hydroxide radical, leaving the monosubstituted product **11b**.

### 3.3. Doebner–Von Miller Reaction

The Doebner–Von Miller (DVM) reaction, an offshoot of the Skraup reaction in which an α,β-unsaturated carbonyl compound replaces the glycerol component, is a commonly-used route toward 2-substituted quinolines. The original method was prone to acid-catalyzed polymerization of the carbonyl substrate, thereby resulting in low yields of product. Therefore the advent of a biphasic reaction medium was a boon to this reaction; sequestering the carbonyl compound in an organic phase drastically reduced polymerization and increased yield [17]. However, the use of this method in the formation of heteroring-unsubstituted quinolines remained unprecedented.

**Figure 9:**
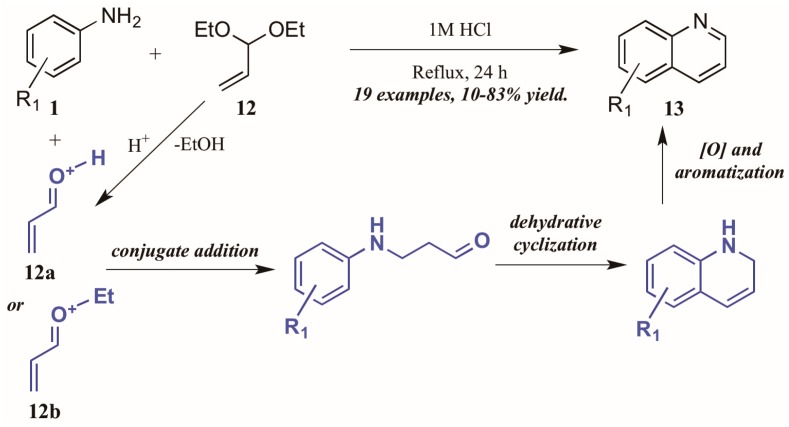
Doebner–Von Miller cyclization of anilines with acrolein diethyl acetal.

Ramann and Cowen [18] described the synthesis of heteroring-unsubstituted quinolines through a modified Doebner–Von Miller route. After initial trials using acrolein in a biphasic reaction medium, they found acrolein diethyl acetal to be a superior annulation partner (Figure 9). Interestingly, they found a biphasic solvent mixture detrimental to the efficiency of the reaction; performing the reaction solely in dilute HCl improved the outcome significantly, resulting in quinolines with a wide variety of substitution patterns in fair to excellent yields. The reaction is presumed to proceed through a typical Skraup–DVM mechanism [19], with the aniline performing conjugate addition to either protonated acrolein or an oxocarbenium ion, both of which are possible acid-mediated hydrolysis products of acrolein diethyl acetal. Dehydrative ring closure, followed by oxidative aromatization, results in the final quinoline products.

### 3.4. Conrad–Limpach Reaction

Aryl amines and β-ketoesters are combined under thermal conditions to form quinoline-4-ols in the Conrad–Limpach reaction. Although this is considered a universal process [20], it presents several challenges. The original solvent-free method was relatively inefficient, however the high-energy imine-enol intermediate compulsory for cyclization necessitated any solvent added to the mixture to be high-boiling. The mineral oil, diphenyl ether, and Dowtherm A commonly used as solvents are either prohibitively expensive or difficult to remove from the product [21].

**Figure 10:**
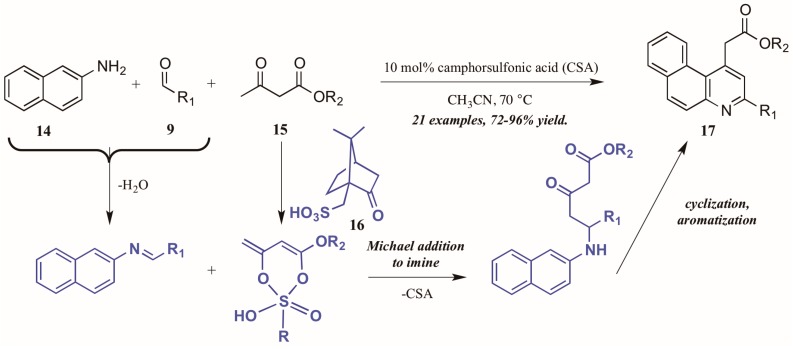
Acid-catalyzed multicomponent Conrad–Limpach reaction.

Khan and coworkers [22] approached the synthesis of biologically-relevant 4-acetylquinolines through a Conrad–Limpach-like multicomponent reaction (MCR). As an atom-economical way to combine three or more molecules, MCRs are a powerful tool in the construction of complex structures [23]; this tactic was utilized in the formation of quinolines via naphthylamine (**14**, Figure 10), aldehyde (**9**), and β-ketoester (**15**) components under camphorsulfonic acid (CSA, **16**) catalysis. The reaction is presumed to proceed through a Michael-like γ-addition of the CSA-activated β-ketoester to the preformed imine. The resultant intermediate then cyclizes and aromatizes to give the product quinolines **17** in excellent yield. Notably, by employing this MCR approach, they avoided the high-energy intermediate common to this type of reaction and thus were able to perform the reaction in low-boiling acetonitrile.

### 3.5. Povarov Reaction

As previously mentioned, multicomponent reactions make it possible to construct complex compounds in a single atom-economical step. The Povarov reaction is an example of an established aza-heterocycle synthesis via MCR. Typically, the Povarov reaction proceeds via the inverse electron-demand Diels–Alder reaction of an aryl amine, aldehyde, and activated alkene to form tetrahydroquinolines.

**Figure 11:**
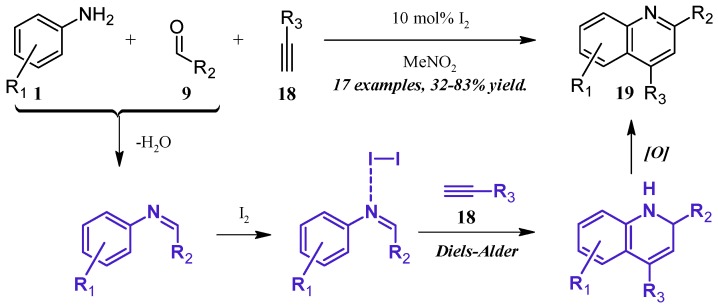
Iodine-catalyzed multicomponent Povarov reaction.

Lin et al. [24] reported a molecular iodine-catalyzed version of the Povarov reaction (Figure 11), in which quinoline derivatives were obtained directly from aniline (**1**), aldehyde (**9**), and alkyne (**18**) precursors. The aniline **1** first forms a Schiff base with the aldehyde **9**, which then coordinates with the Lewis acidic I_2_. This complex then regioselectively goes through a Diels–Alder cycloaddition with the alkyne **18**, forming a dihydro intermediate, which is oxidized by the ambient atmosphere to give the 2- and 4-substituted quinoline **19** in fair to good yield.

**Figure 12:**
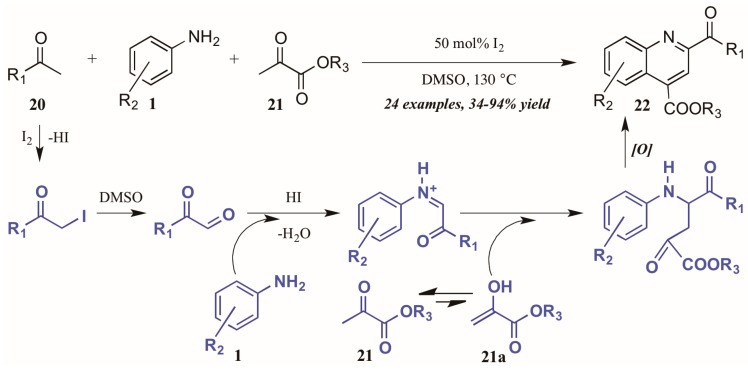
Iodine-catalyzed Povarov reaction.

Wu and coworkers [25] recently described another I_2_-catalyzed Povarov-type reaction, in which they envisioned a more complex role for iodine in the presence of arylamines, methylketones, and α-ketoesters (Figure 12). They propose addition of iodine from I_2_ to the alpha-carbon of methyl ketone **20**. The solvent DMSO then oxidizes the α-iodo ketone, which is subsequently attacked by the aniline **1**. The resultant C-acyliminium reacts with the ketoester enol tautomer **21a**. Electrophilic cyclization, followed by oxidative aromatization, gives the 2,4-disubstituted product **22**.

## 4. Quinoline Synthesis via Modifications to Non-Aniline-Based Established Methods

### 4.1. Pfitzinger Reaction

The Pfitzinger reaction is a divergent approach to the synthesis of 2-substituted quinoline-4-carboxylic acids by the reaction of isatin with ketones in the presence of a strong nucleophile (typically hydroxide). Pfitzinger’s own studies of the late nineteenth century indicate this reaction is generally efficient-he reported obtaining 2-methylquinoline-4-carboxylic acid in up to 80% yield [26]. However, there are also less-efficient examples of this reaction, owing to a thick resin surrounding the crude product and hampering isolation [27]. Recent research on this reaction documents novel substitutions on the quinoline system.

**Figure 13:**
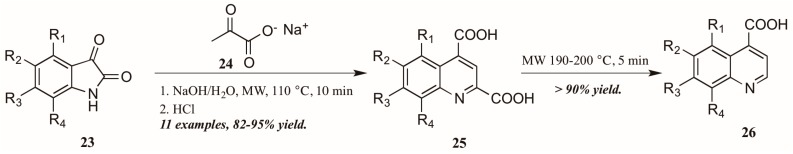
Pfitzinger reaction under microwave irradiation with selective decarboxylation.

Zhu and coworkers [28], noting the dearth of methods to obtain quinoline-4-carboxylic acids unsubstituted in the 2-position, utilized the Pfitzinger reaction in combination with selective decarboxylation to obtain these analogs (Figure 13). Reacting isatin **23** with sodium pyruvate **24** in an aqueous solution of sodium hydroxide under microwave irradiation, they were able to obtain the desired quinoline-2,4-dicarboxylic acids **25**. These were then subjected to short microwave irradiation at 190–200 °C to selectively decarboxylate at the 2-position, leaving the quinoline-4-carboxylic acids **26** in overall very good yield.

**Figure 14:**
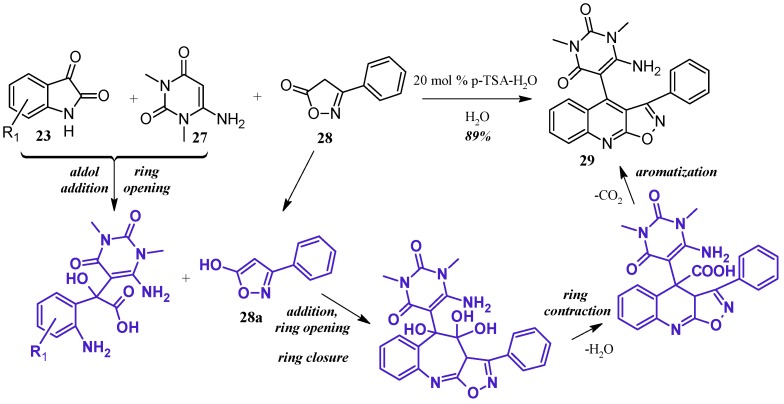
Acid-catalyzed multicomponent Pfitzinger reaction.

Perumal et al. [29] achieved aminouracil-substituted isoxazolsquinolines through a Pfitzinger multicomponent reaction (Figure 14). Under a catalytic amount of *p*-toluenesulfonic acid, isatin **23** and 6-aminouracil **27** undergo aldol addition and ring opening to give isatic acid. The enol tautomer of isoxazole **28a** attacks the isatinic acid, and attack of the amine on isoxazole carbonyl forms a seven-member ring. With loss of H_2_O, the ring contracts, and decarboxylation aromatizes the ring, giving the final quinoline product **29** in excellent yield.

**Figure 15:**
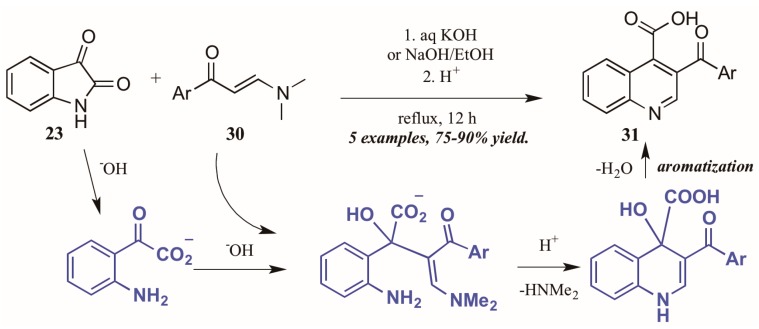
Pfitzinger reaction using enaminones under basic conditions.

Elghamry and Al-Faiyz [30] investigated the use of enaminones as the annulation partner in the Pfitzinger synthesis of 3-aroylketone quinoline-4-carboxylic acids (Figure 15). In aqueous sodium or potassium hydroxide, the isatin (**23**) ring opens, giving isatinate. Aldol-like addition of the alpha carbon of the enaminone **30** to isatinate gives an intermediate, which cyclizes under acidic conditions. Loss of H_2_O gives the aromatized product **31** in excellent yield.

### 4.2. Friedländer Reaction

Among the many established methods of quinoline synthesis, the Friedländer reaction is one of the oldest, and one of the simplest. Its popularity stems mainly from its versatility; many functional groups are well tolerated on both the arylamine and ketone components. However, this reaction is not without its drawbacks. For instance, regioselectivity is difficult to control when unsymmetrical ketone annulation partners are used, resulting in the formation of both 2-substituted and 2,3-disubstituted products [31]. Therefore, several groups have recently reported modified procedures of this venerable method.

**Figure 16:**
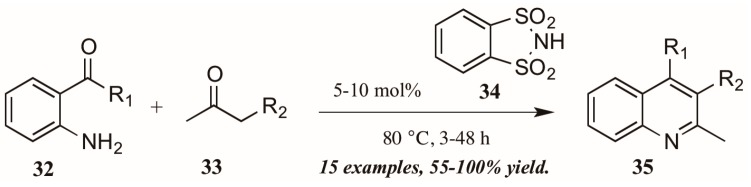
Brønsted acid-mediated Friedländer quinoline synthesis.

Several versions of Brønsted acid-mediated Friedländer quinoline synthesis have appeared in the literature in the last few years. One example was reported by Dughera and coworkers [32], who had previously utilized the acid catalyst *o*-benzenedisulfonimide (**34**, Figure 16) in several syntheses and found it to be non-corrosive, non-volatile, and recyclable. They applied this acid catalyst to a solvent-free Friedländer reaction of 2-aminoarylketones (**32**) with various carbonyl compounds (**33**). In this way, they obtained several 2,3-disubstituted quinolines (**35**), including fused five-member rings, with good to excellent yields.

**Figure 17:**
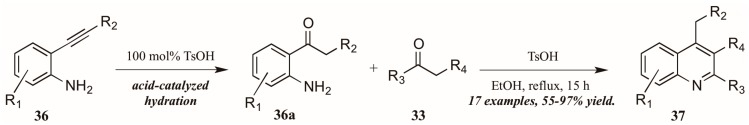
Brønsted acid-mediated Friedländer reaction starting from 2-alkynylanilines.

Zhu et al. [33] used *p*-toluenesulfonic acid as a catalyst in their pursuit of elusive 4-alkylquinolines (**37**, Figure 17). Refluxing the carbonyl component **33** with 2-alkynylanilines **36** in ethanol initially resulted in acid-mediated hydration of the alkyne to the corresponding ketones **36a**. These then cyclized in a typical Friedländer fashion, giving the desired 4-alkylquinolines **37** in moderate to very good yield.

**Figure 18:**
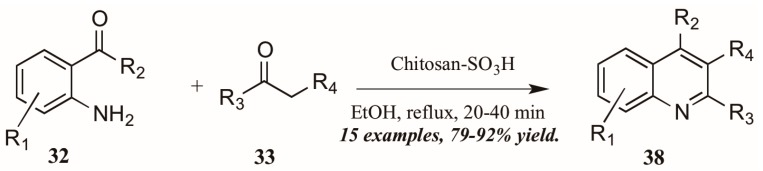
Chitosan-SO_3_H-catalyzed Friedländer quinoline synthesis.

Reddy et al. [34] also investigated Brønsted acid catalysts in Friedländer reactions. They found the solid acid Chitosan-SO_3_H (Figure 18) to be a highly efficient catalyst in the annulation of 2-aminoarylketones **32** with various carbonyl compounds **33**, including cyclic and sterically hindered examples to give diverse 2,3,4-trisubstituted quinolines **38** in very good yield after short reflux times. In addition, they were gratified to find that the solid acid was easily recovered from the reaction mixture, reactivated, and reused with comparable success.

**Figure 19:**
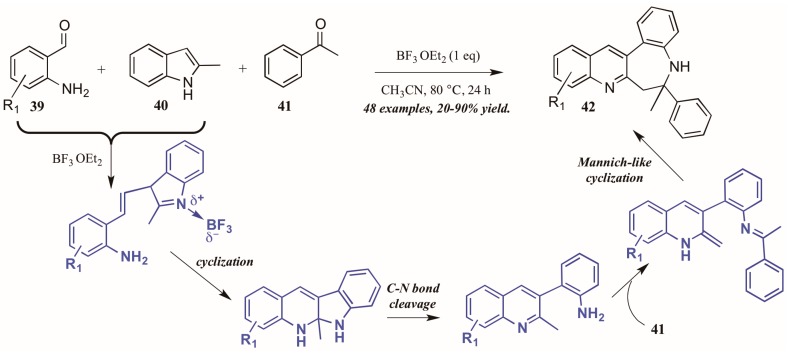
Lewis acid-catalyzed Friedländer quinoline synthesis.

Additionally, Lewis acid catalysis has featured in recent literature on the Friedländer quinoline synthesis. Gu and coworkers [35] reported the Lewis acid-catalyzed formation of unexpected benzazepine-fused quinoline products from 2-aminobenzaldehydes, 2-methylindole, and acetophenone (Figure 19). The proposed mechanism begins with Lewis acid-mediated combination of 2-aminobenzaldehyde **39** with 2-methylindole **40**. This intermediate then goes through nucleophilic cyclization, followed by C-N bond cleavage. The free arylamine then attacks acetophenone **41**, forming an adduct, which then undergoes an enamine-activated Mannich-like cyclization to form the benzazepine-fused quinolines **42** in fair to excellent yields.

**Figure 20:**
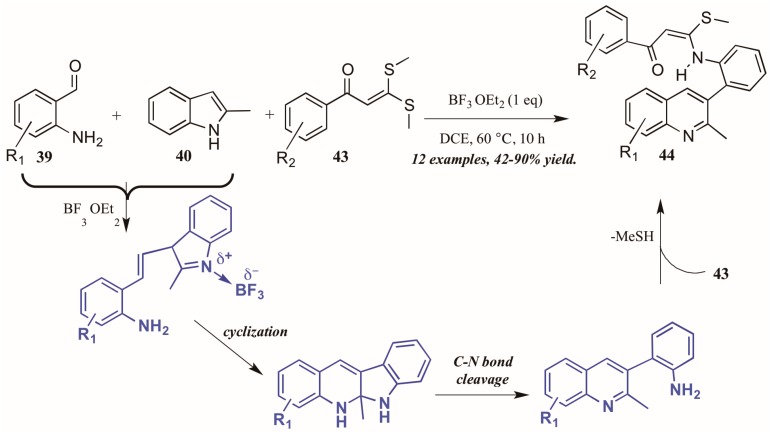
Lewis acid-catalyzed multicomponent Friedländer quinoline synthesis with dithioethers.

Guo et al. found similarly unforeseen success with a related Lewis acid-catalyzed multicomponent reaction of 2-aminobenzaldehydes and 2-methylindole. However, the third component, bis(methylsulfanyl) phenylpropenone, showed divergent reactivity as compared to acetophenone (Figure 20). The reaction is presumed to proceed through a similar mechanism, beginning with the BF_3_-mediated coupling of 2-aminoacetophenone **39** and 2-methylindole **40**. Then, after cyclization and C-N bond cleavage, the arylamine attacks bis(methylsulfanyl) phenylpropenone **43**, extruding methylsulfide and forming the final quinoline product **44** in good to excellent yield.

**Figure 21:**
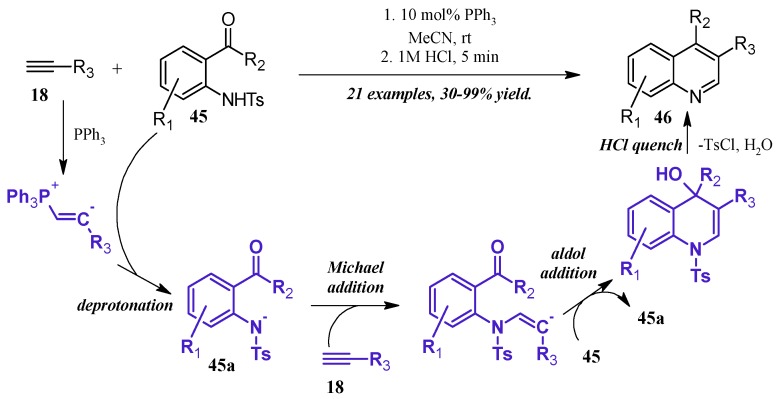
Phosphine-catalyzed Friedländer quinoline synthesis.

Lewis bases can also play a catalytic role in the synthesis of quinolines. Kwon et al. [36] reported a robust phosphine-catalyzed annulation of *N*-tosylated 2-aminoaryl aldehydes and ketones with alkynes to give dihydroquinolines, which were easily converted to the quinoline by a quench with dilute HCl (Figure 21). Kwon proposed three potential mechanisms for the reaction and attempted to positively identify the correct pathway by pinpointing key intermediates via NMR studies. While this was ultimately unfruitful, they determined the reaction to most likely proceed through a general base catalysis mechanism. The alkyne **18** is first activated by the phosphine and subsequently deprotonates the amine **45**, which is rendered more acidic due to the tosyl group. The amine nucleophile **45a** then adds to a second alkyne molecule, which cyclizes, deprotonates another tosylated amine, then aromatizes under acidic quench to the product quinoline **46**.

**Figure 22:**
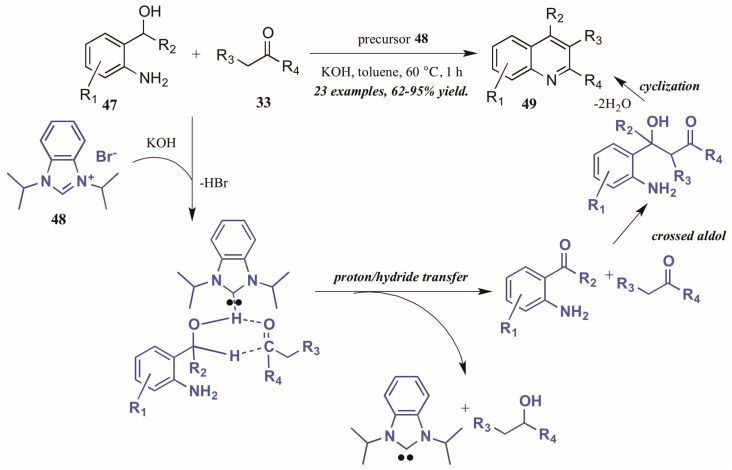
N-Heterocyclic carbene-catalyzed Friedländer quinoline synthesis.

Zhu and Cai [37] used N-heterocyclic carbenes (NHCs), another type of Lewis base, to obtain diversely substituted quinolines from 2-aminobenzyl alcohols and ketones (Figure 22). They proposed the NHC-generated in situ from deprotonation of the precursor **48** by hydroxide-may assist in the tandem proton and hydride transfer from the benzyl alcohol **47** to the ketone **33**. This creates the active intermediates, which undergo crossed aldol addition and then cyclize with loss of H_2_O to give the final quinoline products **49** in good to excellent yield.

**Figure 23:**
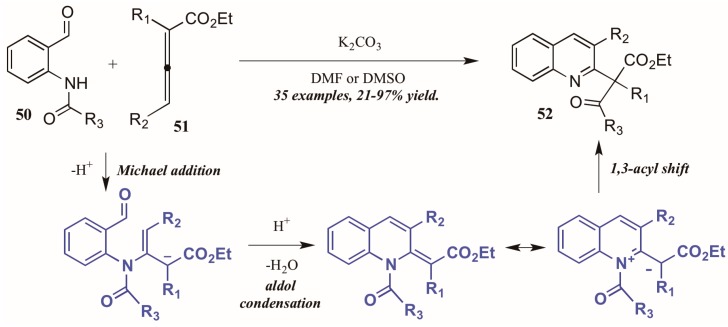
Base-mediated cyclization of 2-aminobenzaldehydes with allenoates.

Citing the inherent electrophilic and nucleophilic character of allenoates (α-allenic esters), Selig and Raven [38] investigated the use of these compounds in the formation of quinolines. Although their initial trials using *o*-aminobenzaldehyde did not result in the cyclization product, they found that monoprotection of the amine allowed the process to occur. Interestingly, they observed formation of both the desired quinoline product as well as a second product that featured a migration of the protecting group and formation of a new quaternary center (Figure 23). They propose the mechanism to operate through an aza-Michael addition of the Brønsted base-promoted amide anion of the 2-aminobenzaldehyde **50** to the β carbon of the allenoate **51**, which then cyclizes selectively from the γ position. The resultant intermediate can then be depicted in its zwitterionic form, from which it performs a 1,3-shift of the protecting group to give the product. Under optimized reaction conditions, they were able to selectively synthesize the rearrangement products **52** in high yields using various protecting groups.

**Figure 24:**
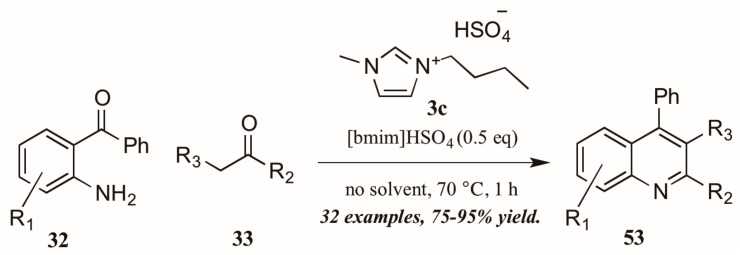
Ionic liquid-catalyzed Friedländer quinoline synthesis.

As with the previously mentioned Skraup and Doebner reactions, the Friedländer quinoline synthesis can be improved by the use of ionic liquids as either solvent or catalyst. Tajik and coworkers [39] reported an efficient quinoline synthesis using substoichiometric amounts of the ionic liquid [bmim]HSO_4_ (**3c**, Figure 24). The short reaction time, high yield, and solvent-free conditions recommend this procedure as a green alternative to traditional Friedländer reactions.

**Figure 25:**
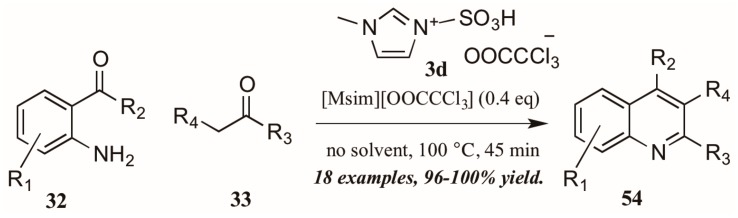
Ionic liquid acid-catalyzed Friedländer quinoline synthesis.

Sarma et al. [40] also investigated the use of Brønsted-acidic ionic liquids as catalysts in a classical Friedländer reaction (Figure 25). They found their novel ionic liquid catalyst [Msim][OOCCCl_3_] **3d** to be at least as efficient as other known ionic liquids and acid catalysts, producing quinolines in yields up to 100%. In addition, the ability to recycle and reuse this catalyst was particularly appealing.

**Figure 26:**
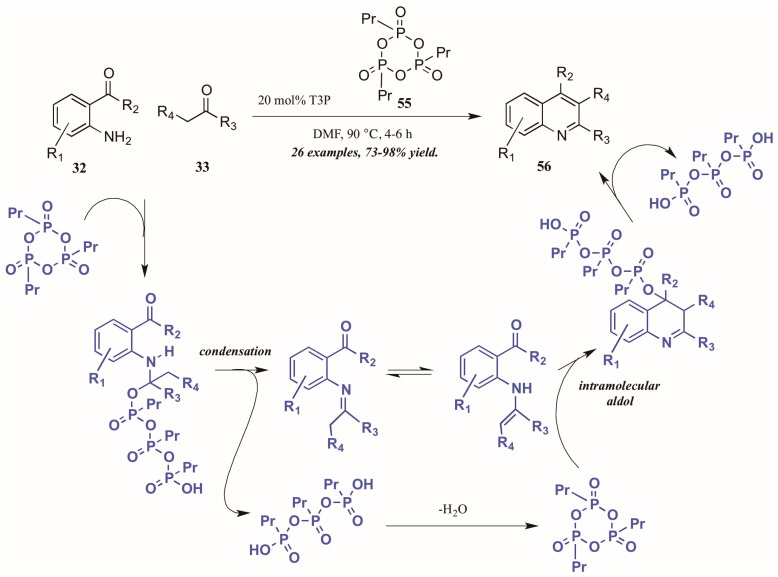
Friedländer quinoline synthesis via peptide coupling reagent.

Citing its recent application to the preparation of heterocycles, Augustine and coworkers explored the use of peptide coupling agent propylphosphonic anhydride (T3P, **55**, Figure 26) in the Friedländer reaction. They were gratified to find that a catalytic loading of T3P to the reaction was sufficient to give very high yields of diversely-substituted quinolines. Mechanistically, they propose T3P to assist in the initial condensation of **32** and **33**, as well as the subsequent intramolecular aldol condensation to form the final product **56**.

**Figure 27:**
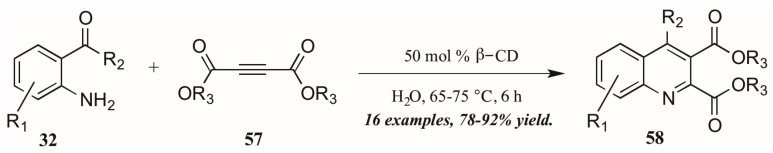
Friedländer quinoline synthesis via biomimetic catalysis.

As a final example of a novel approach to the Friedländer quinoline synthesis, Nageswar and coworkers employed a biomimetic method in which β-cyclodextrin acts in enzyme-like fashion, performing catalysis of the reaction within its hydrophobic cavity (Figure 27). In this way, reactions involving hydrophobic substrates can be run with water as the sole solvent. With a catalytic amount of β-CD, they obtained various quinoline-2,3-dicarboxylates **58** under neutral aqueous conditions in excellent yield, with a high recovery of catalyst.

## 5. Quinoline Synthesis via Novel Synthetic Routes

### 5.1. Electrophilic Cyclization

**Figure 28:**
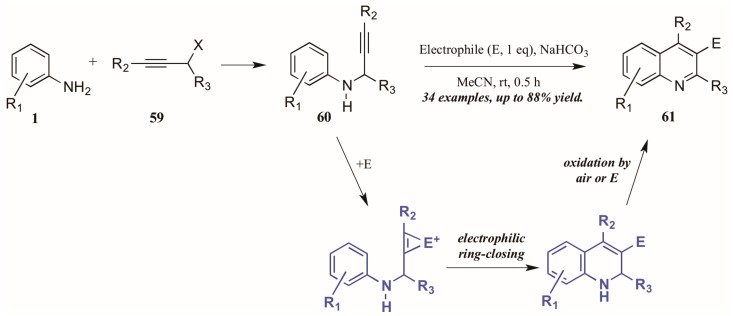
Quinoline synthesis via electrophilic cyclization of anilines and alkynes.

Larock and coworkers [41] described the first quinoline synthesis via electrophilic cyclization in 2010. Envisioning a means of forming quinolines which contain functional groups that may not be amenable to any of the established methods, they investigated an electrophile-induced cyclization reaction (Figure 28). The first step involved formation of propargylic arylamines **60** from anilines **1** and haloalkynes **59**, which were then treated with the electrophile-ICl, I_2_, Br_2_, PhSeBr, or ArSCl. It was conjectured that the electrophile adds to the triple bond, which enhances the electrophilicity of the alkyne, activating it toward attack by the aromatic ring. Through oxidation by air or the electrophile, the ring is aromatized, giving 3-halogen, selenium, or sulfur-substituted quinolines **61** in poor to very good yields.

**Figure 29:**
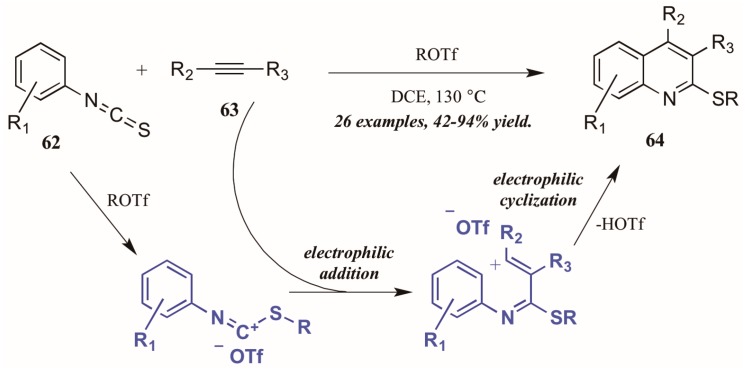
Alkyltriflate-mediated electrophilic cyclization of isothiocyanates with alkynes.

Xi et al. [42] reported alkyltriflate-mediated electrophilic cyclization of arylisothiocyanates **63** and alkynes (**62**, Figure 29). The reaction was presumed to proceed through an alkyltriflate-formed carbenium ion, to which the alkyne adds regioselectively. The intermediate cyclizes, with triflate assisting in the necessary deprotonation and aromatization. In this way, they were able to obtain various 2-thioquinolines **64** in good to excellent yield.

**Figure 30:**
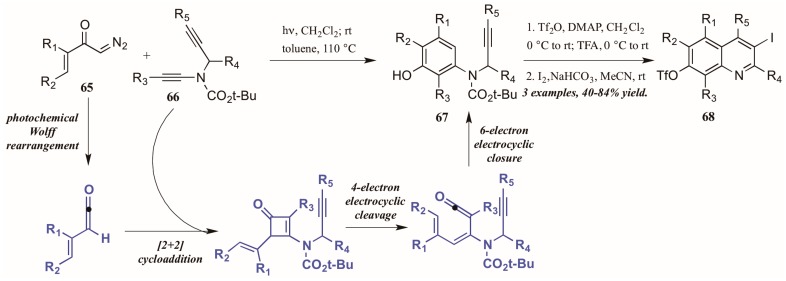
Photochemical electrophilic cyclization to form highly functionalized quinolines.

Danheiser and coworkers [43] developed an elaborate two-stage benzannulation/iodocyclization strategy toward highly substituted quinolines (Figure 30). The first stage involves the photochemical Wolff rearrangement of α,β-unsaturated α-diazo ketones **65** to transient vinylketenes. The ketene is immediately involved in a regioselective [2 + 2] cycloaddition with an ynamide **66**, giving an intermediate cyclobutenone. After four-electron electrocyclic cleavage of the butene ring, six-electron electrocyclic closure forms the aminophenol ring (**67**). Upon introduction of an electrophile such as I_2_, the alkyne appendage performs electrophilic cyclization with the aromatic ring, affording quinolines with multiple functionalities (**68**).

**Figure 31:**
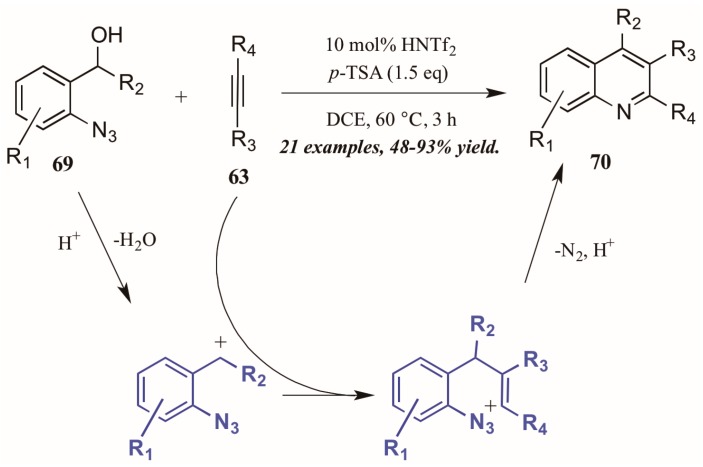
Acid-mediated cyclization of arylazides with alkynes.

Stopka and Niggemann [44] recently disclosed the formation of substituted quinolines through annulation of 2-azidobenzyl alcohols with internal alkynes under acidic conditions (Figure 31). The reaction proceeds through an acid-catalyzed dehydration of alcohol **69** to form a benzylic cation. This cation readily reacts with the alkyne **63**, forming a vinyl cation. The nearby azide group attacks this cation, and with loss of N_2_ and deprotonation, the quinoline **70** is formed.

**Figure 32:**
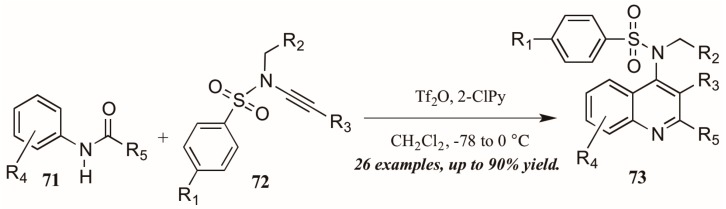
Electrophilic cyclization of aryl amides with sulfanyl ynamides.

Finally, Bräse and coworkers [45] described the synthesis of functionalized quinolines from sulfanyl ynamides **72** and electrophilically-activated aryl amides **71** under the influence of triflic anhydride and 2-chloropyridine (Figure 32). Inspired by the work of Movassaghi, who developed this method to synthesize pyridine, pyrimidine, and β-carboline derivatives, the Bräse group applied this procedure to quinoline synthesis. They obtained many 4-sulfonylamino quinolines (**73**) in good yield, which could then be easily converted to biologically useful amines by hydrolysis of the protecting group.

### 5.2. Oxidative Cyclization

**Figure 33:**
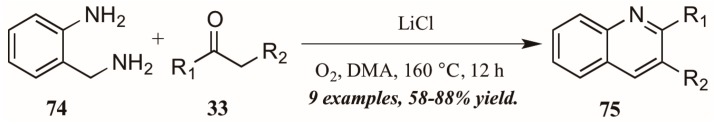
Oxidative cyclization of aryl diamines with carbonyl compounds.

As efforts to perform chemical syntheses in environmentally friendly ways has gained momentum in recent years, considerable interest has been given to aerobic oxidation as a method of bond activation. Lei and coworkers [46] applied this method to quinoline synthesis using simple primary amines (Figure 33). In fact, when 2-(aminomethyl)aniline **74** was reacted with various aryl ketones **33** under an oxygen atmosphere, the corresponding 2-aryl quinolines **75** were obtained in good yield.

**Figure 34:**
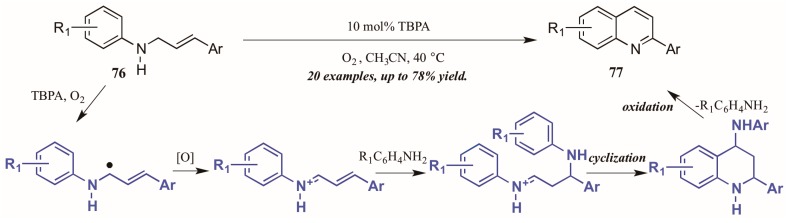
Radical cation-catalyzed intramolecular oxidative cyclization.

Jia et al. [47] also used molecular oxygen, in conjunction with the radical cation salt tris(4-bromophenyl)aminium hexachloroantimonate (TBPA), to induce oxidation of sp^3^ C-H bonds adjacent to nitrogen (Figure 34). Reacting substituted *N*-cinnamylanilines under O_2_ atmosphere with a catalytic amount of the radical cation salt, they achieved various 2-aryl quinolines. They propose the initial step of the mechanism to be oxidation of the C-H bond next to nitrogen by TBPA and O_2_ to give the radical intermediate, which is then further oxidized to give the iminium ion. This is then attacked by the aniline extruded from TBPA via Michael-like addition. After cyclization, the aniline is extruded as the ring aromatizes, giving the final product.

**Figure 35:**
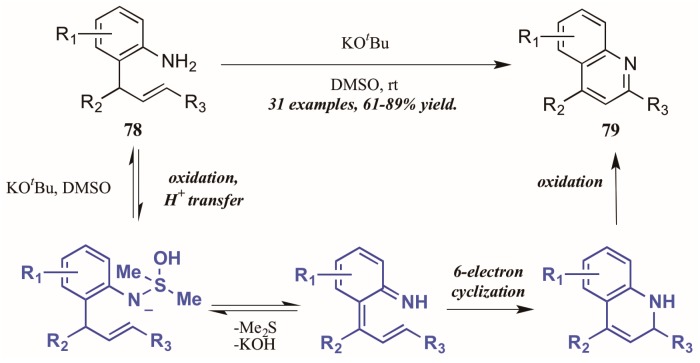
DMSO-mediated oxidative cycloisomerization.

Drawing on previous success forming functionalized indoles under aerobic oxidation and palladium catalysis, Ghorai et al. [48] developed a metal-free cycloisomerization strategy of quinoline synthesis, in which the oxidation is performed by dimethyl sulfoxide (DMSO) rather than molecular oxygen (Figure 35). Reacting preformed *ortho*-allylanilines with a substoichiometric amount of potassium *tert*-butoxide in DMSO, they obtained diverse quinoline products, including 2-styryl derivatives for which a dearth of synthetic options exist. The mechanism is presumed to proceed through oxidation of 2-allylaniline via KO^*t*^Bu and DMSO, then six-electron cyclization to give the dihydroquinoline. This is then oxidized to give the quinoline.

### 5.3. Aza-Wittig Cascade Reactions

**Figure 36:**
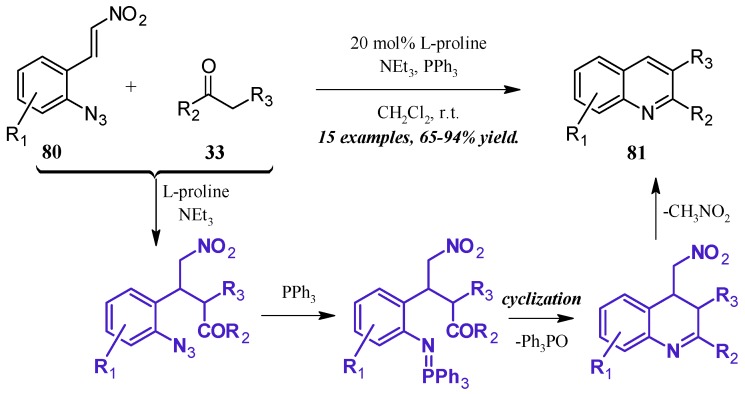
Base-catalyzed aza-Wittig cyclization of quinoines.

The formation of iminophosphoranes, and their subsequent use in aza-Wittig reactions, has become a popular method of nitrogen-heterocycle formation. Shi et al. [49] reported a one-pot, l-proline-catalyzed aza-Wittig cascade reaction resulting in 2- and 3-substituted quinolines (Figure 36). The cascade begins with the Michael addition of the ketone **33** to the β-nitroolefin **80**. A Staudinger reaction of the azide with triphenylphosphine forms the iminophosphorane, which then undergoes aza-Wittig to close the ring. The ring is aromatized with expulsion of nitromethane to give the product quinolines **81** in very good yield.

**Figure 37:**
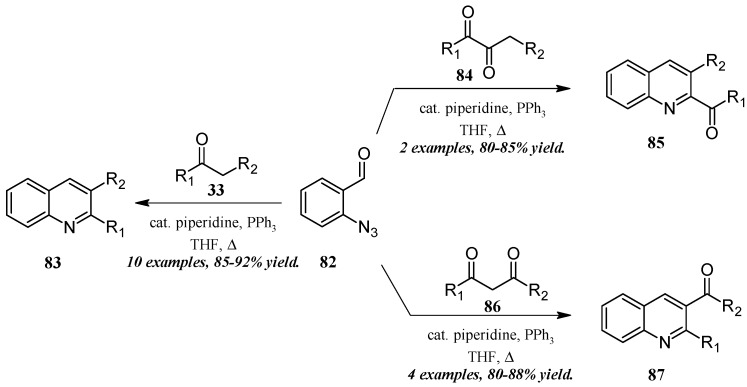
Aza-Wittig cascade reaction of azidobenzaldehyde with mono- and dicarbonyl compounds.

He and coworkers [50] took a similar approach with a cascade reaction of *ortho*-azidobenaldehyde and various carbonyl and dicarbonyl compounds (Figure 37). Through a Knoevenagel/Staudinger/aza-Wittig cascade, this group obtained quinolines in which the 2- and 3-substituents could be altered based on the annulation partner and reaction conditions used. Reacting 2-azidobenzaldehyde **82** with mono-carbonyl compounds **33**, disubstituted quinolines **83** were produced in very good yield. On the other hand, 2-carbonylquinolines **85** were obtained in good yield by reaction with 2,3-carbonyl compounds **84**, while 3-carbonylquinolines **87** were obtained by reaction with 1,3-carbonyl compounds **86**.

### 5.4. Other (Radical-Promoted, Cycloaddition, I_2_ Catalyzed)

**Figure 38:**
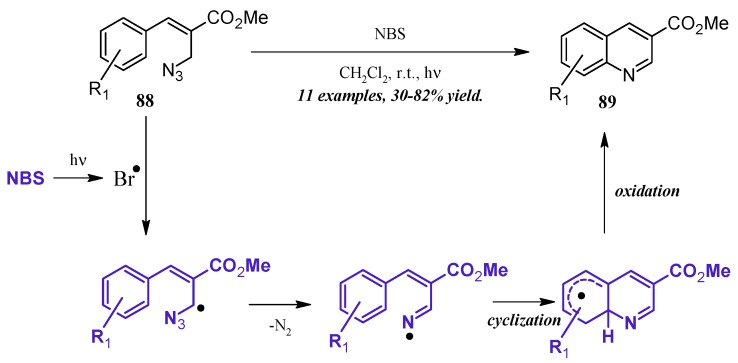
NBS-mediated radical cyclization to form 3-substituted quinolines.

An alternative method of quinoline synthesis involves radical-promoted cyclization of arylamine precursors. Yu and coworkers [51] synthesized quinolines substituted at the 3-position via an *N*-bromosuccinamide-mediated radical reaction (Figure 38). They proposed the mechanism to begin with the visible light-promoted formation of bromine radical from NBS. This radical reacts with the C-H adjacent to the azide on propenoate **88**, and through extrusion of N_2_, the radical imine cyclizing with the aryl ring. Oxidation rearomatizes the ring, giving the desired quinoline products **89** in fair to good yield.

**Figure 39:**
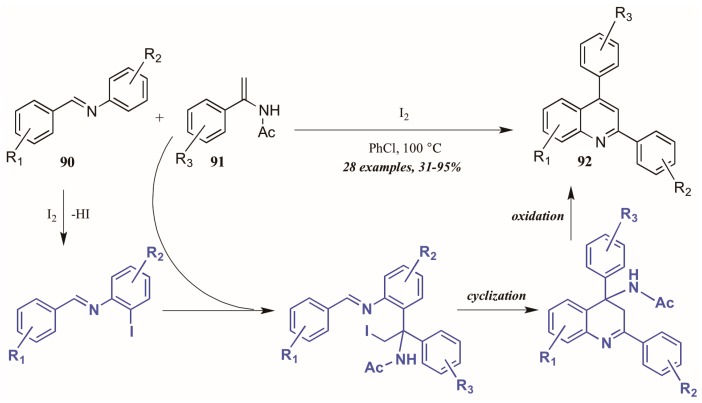
Iodine-catalyzed synthesis of diarylquinolines from enamides and imines.

Huang et al. [52] developed a divergent method of I_2_-catalyzed quinoline synthesis from enamides and imines (Figure 39). The reaction is initiated by *ortho*-iodination of the aryl imine **90**, followed by insertion of the enamide **91** into the newly formed C-I bond. Cyclization and loss of acetamide then form the diaryl quinoline **92**.

**Figure 40:**
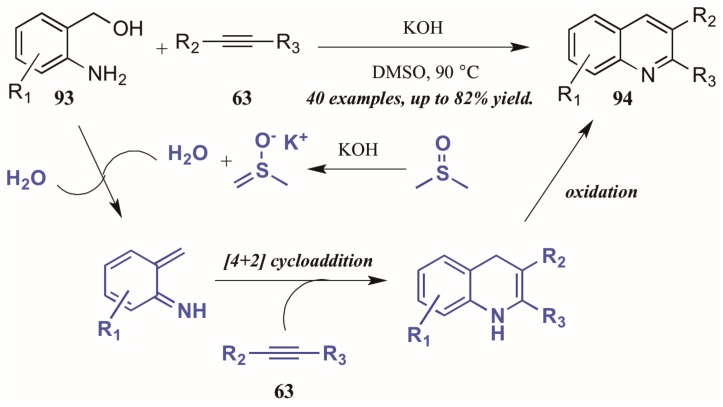
[4 + 2] Cycloaddition of azadienes with internal alkynes.

Verma et al. [53] described a chemo- and regioselective [4 + 2]-cycloaddition of alkynes with in situ-generated azadienes (Figure 40). The mechanism is presumed to initiate with the DMSO/KOH-promoted dehydration of 2-aminobenzyl alcohol **93**, forming the azadiene which was isolated under control experiments. The azadiene then cyclizes with the alkyne **63**, and the quinoline **94** is formed upon oxidation.

**Figure 41:**
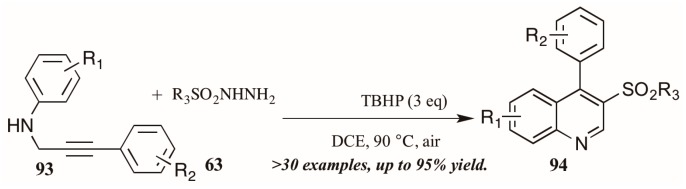
Oxidative cyclization to provide 3-arylsulfonylquinolines.

Finally, a very recent contribution from Tang et al. [54] describes the synthesis of 3-arylsulfonylquinolines **95** from *N*-propargyl aromatic amines **96** and arylsulfonylhydrazides **97** (Figure 41). The oxidative cyclization reaction is promoted by *tert*-butyl hydroperoxide (TBHP) and a single electron mechanistic pathway is proposed to deliver the products in good to excellent yields.

## 6. Conclusions

The quinoline ring system is ubiquitous in the fields of medicinal and industrial chemistry. Because of the broad range of uses for this scaffold, methods aimed at improving the efficiency and versatility of quinoline synthesis have been studied for over a century. Several recent developments in this field involve modifications to established methods such as the Skraup, Doebner–Von Miller, and Friedländer synthetic routes, including the use of ionic liquids, various catalysts, and novel reagents. In addition, examples of innovative processes toward these molecules include oxidative and radical-promoted cyclizations, cascade reactions, and electrophilic annulations. The scope of reactivity presented in this review indicates the availability of efficient, practical, metal-free synthetic routes toward quinolines with nearly any substitution pattern desired.
